# Inhibition of *α*-Synuclein Accumulation Improves Neuronal Apoptosis and Delayed Postoperative Cognitive Recovery in Aged Mice

**DOI:** 10.1155/2021/5572899

**Published:** 2021-05-28

**Authors:** Yue Li, Yi Yuan, Yitong Li, Dengyang Han, Taotao Liu, Ning Yang, Xinning Mi, Jingshu Hong, Kaixi Liu, Yanan Song, Jindan He, Yang Zhou, Yongzheng Han, Chengmei Shi, Shun Yu, Peng Zou, Xiangyang Guo, Zhengqian Li

**Affiliations:** ^1^Department of Anesthesiology, Peking University Third Hospital, Beijing 100191, China; ^2^Department of Anesthesiology, Beijing Jishuitan Hospital, Beijing 100035, China; ^3^Department of Neurobiology, Beijing Institute of Geriatrics, Xuanwu Hospital, Capital Medical University, Beijing, China; ^4^Center of Parkinson's Disease, Beijing Institute for Brain Disorders, Beijing, China; ^5^Beijing Key Laboratory for Parkinson's Disease, Beijing 100053, China; ^6^College of Chemistry and Molecular Engineering, Synthetic and Functional Biomolecules Center, Beijing National Laboratory for Molecular Sciences, Key Laboratory of Bioorganic Chemistry and Molecular Engineering of Ministry of Education, Peking University, Beijing 100871, China

## Abstract

Delayed neurocognitive recovery (dNCR) is a major complication after anesthesia and surgery in older adults. Alpha-synuclein (*α*-syn; encoded by the gene, *SNCA*) has recently been shown to play an important role in hippocampus-dependent working memory. Aggregated forms of *α*-syn are associated with multiple neurotoxic mechanisms, such as mitochondrial dysfunction and cell death. In this study, we found that blocking *α*-syn improved both mitochondrial function and mitochondria-dependent neuronal apoptosis in a mouse model of dNCR. Various forms of *α*-syn (including total *α*-syn, phosphorylated-Ser129-*α*-syn, and oligomers) were upregulated in hippocampal tissue and extracted mitochondria after surgical challenge. Clenbuterol is a novel transcription modulator of *Scna*. Clenbuterol significantly attenuated surgery-induced progressive accumulation of various toxic *α*-syn forms in the hippocampus, as well as mitochondrial damage and memory deficits in aged mice following surgery. We also observed excessive mitochondrial *α*-syn accumulation and increased mitochondria-mediated apoptosis *in vitro* using nerve growth factor-differentiated PC12 cells and primary hippocampal neurons exposed to lipopolysaccharide. To further validate the neuroprotective effect of *α*-syn inhibition, we used a lentiviral *Snca*-shRNA (Lv-shSnca) to knockdown *Snca*. Of note, Lv-shSnca transfection significantly inhibited neuronal apoptosis mediated by the mitochondrial apoptosis pathway in neurons exposed to lipopolysaccharide. This *α*-syn inhibition improved the disruption to mitochondrial morphology and function, as well as decreased levels of apoptosis. Our results suggest that targeting pathological *α*-syn may achieve neuroprotection through regulation of mitochondrial homeostasis and suppression of apoptosis in the aged hippocampus, further strengthening the therapeutic potential of targeting *α*-syn for dNCR.

## 1. Introduction

Delayed neurocognitive recovery (dNCR) describes cognitive decline with symptoms diagnosed up to 30 days following anesthesia and surgery; it is the most common complication in older patients [1]. Importantly, dNCR can last up to several years, potentially promoting the risk of Alzheimer's disease and early mortality, ultimately leading to an economic burden on healthcare resources [2, 3]. However, the pathogenesis of dNCR remains unclear.

Mitochondrial dysfunction has long been recognized as a key factor in the progression of neurodegenerative diseases [4]. Prevention of mitochondrial dysfunction is suggested to be an effective strategy for preventing cognitive impairment [5, 6]. Previous studies have shown that dNCR triggers mitochondrial damage, including a decreased mitochondrial membrane potential, oxidative damage, and mitochondrial respiratory dysfunction [5, 7–9]. To support their normal function and survival under physiological conditions, neuronal cells have a high energy demand, which is supplied by mitochondria. In this regard, understanding the mechanisms for maintaining mitochondrial homeostasis in neuronal cells is essential for clarifying the pathogenesis of dNCR.

Alpha-synuclein (*α*-syn; encoded by the gene, *SNCA*) is a soluble and highly conserved presynaptic unfolded protein. *α*-syn plays a role in regulating synaptic neurotransmitter release and stabilizing complexes of SNARE family proteins, but the detailed mechanisms remain undefined [10, 11]. *α*-syn can misfold and aggregate under various factors, including raised calcium concentration, oxidative stress, gene mutations, and interactions with many other proteins [12]. Aggregation and propagation of misfolded *α*-syn in the brain are involved in the pathogenesis of neurodegenerative diseases, especially in Parkinson's disease cases [13, 14]. In our earlier work, we demonstrated that anesthesia and surgery increased *α*-syn oligomerization and disturbed neurotransmitter homeostasis in the hippocampus of aged rats, while exosome *α*-syn release into the plasma of postoperative delirium (POD) patients with hip fractures was significantly higher compared with non-POD cases [15, 16]. In addition, an earlier study from another laboratory found elevated total *α*-syn expression in the cortex after 12 h, with attention deficit after 24 h in mice following anesthesia plus surgery [17]. Furthermore, Sunwoo et al. reported a significant accumulation of phosphorylated (p)-*α*-syn in the myenteric plexus of patients with POD after gastrectomy [18]. A later study by the same research team reported that Parkinson's disease-related nonmotor symptoms are a marker reflecting the underlying burden of *α*-syn deposition and may act as risk factors for POD in elderly patients [19]. Hence, our studies and others suggest that the accumulation of *α*-syn likely plays a key role in cognitive decline after anesthesia and surgery in humans and mice. More importantly, *α*-syn can translocate to mitochondria [20], especially under stress conditions, although the link between *α*-syn accumulation and mitochondrial pathology of dNCR remains elusive.

Therefore, in this study, we hypothesized that anesthesia and surgical traumas impair mitochondrial function by partially facilitating the progressive accumulation of toxic *α*-syn in aged mice, which results in mitochondria-dependent apoptosis in the hippocampus. To determine whether mitochondrial dysfunction is susceptible to *α*-syn accumulation, we used clenbuterol, a novel modulator of the SNCA gene, to inhibit Snca transcription in aged mice following anesthesia and surgery. Moreover, lentivirus *Snca*-shRNA (Lv-shSnca) was used in transfected nerve growth factor- (NGF-) differentiated PC12 cells and primary hippocampal neurons exposed to lipopolysaccharide (LPS). We found that both *α*-syn expression and mitochondrial activity were impaired *in vivo* and *in vitro*. Furthermore, we attenuated the accumulation of *α*-syn as a proof of principle to determining whether *α*-syn is a viable therapeutic option in preventing progressive cognitive decline and the development of dNCR.

## 2. Material and Methods

### 2.1. Animals

**Figure 1:**
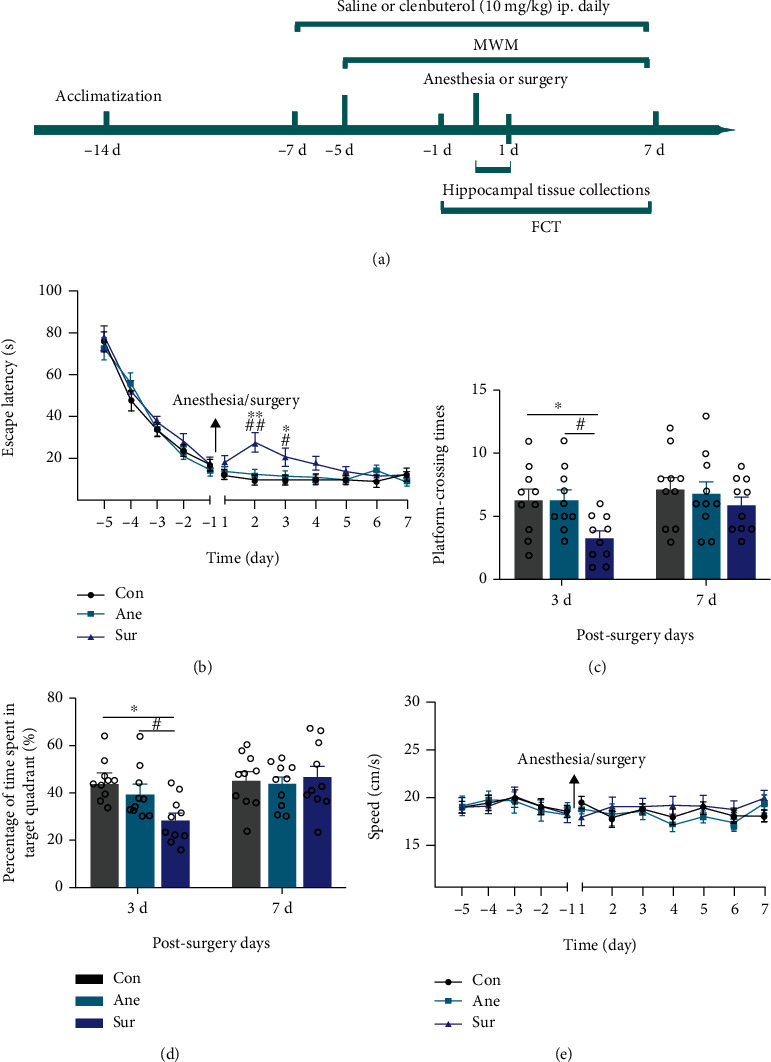
Surgery plus anesthesia impaired spatial cognition in aged mice. (a) Schematic timeline of the experimental protocol. (b) Escape latency in the Morris water maze (MWM) test. (c) Platform crossings during the probe trial of the MWM test. (d) Analysis of time spent in the target quadrant during the probe trial of the MWM. (e) Swimming speed in the MWM training and probe tests. Data are shown as mean ± SEM (*n* = 10 per group) and were compared by repeated measure two-way analysis of variance with post hoc Bonferroni analysis. ^∗^*P* < 0.05, ^∗∗^*P* < 0.01, control (Con) vs. surgery plus anesthesia (Sur); ^#^*P* < 0.05, ^##^*P* < 0.01, anesthesia-alone (Ane) vs. surgery plus anesthesia (Sur).

Aged mice (18 months old, 25–30 g) were obtained from the Experimental Animal Center of Hubei Provincial Academy, Wuhan, China. The mice were reared in a quiet, temperature-controlled room and entrained to a 12 h light/dark cycle with free access to food and water. The experimental protocol was performed in accordance with the Animal Care and Use Committee of Peking University (Beijing, China; Certification number LA20190113). The detailed animal protocol is presented in the schematic diagram (Figure 1(a)).

### 2.2. Anesthesia and Surgery

Mice were randomly placed into the above groups by weight. Mice undergoing the water maze cognitive test were trained for five consecutive days and then randomly assigned to four groups: (1) control group (Con), mice received no intervention; (2) anesthesia-alone group (Ane), mice were anesthetized using 2.5% sevoflurane (in 100% oxygen) in an induction chamber (RWD Life Science, Shenzhen, China) for 5 min. Anesthesia was maintained using 2.5% sevoflurane for a total of 25 min. The mice that breathed spontaneously and sevoflurane concentration were measured continuously (Datex, Tewksbury, MA, USA). A previous study found that the anesthesia protocol does not significantly alter blood gas and blood pressure [21]. Finally, mice recovered on a thermal insulation blanket in a chamber filling with 100% oxygen until they regained consciousness; (3) surgery and anesthesia group (Sur), mice in the surgery group had a simple laparotomy under sevoflurane anesthesia using the methods described in the anesthesia-alone method above [22, 23]. Specifically, a 2 cm longitudinal midline incision was made and rubbed for 30 s. The incision was then closed using 5-0 suture sterile silk sutures and cleaned with iodophor three times followed by 0.25% bupivacaine infiltration. The mice then recovered on a thermal insulation blanket in a chamber containing 100% oxygen. Finally, lidocaine hydrochloride gel was applied to the wound every 8 h to relieve the pain and stress; and (4) surgery plus clenbuterol group (Sur+Clen), mice were treated as in the Sur group and underwent anesthesia plus surgery with the administration of clenbuterol. Clenbuterol treatment (10 mg/kg, Sigma, Poole, UK) by intraperitoneal injection started 7 days before the operation, and injection of clenbuterol continued daily until the end of the experiment (day 3 and day 7 after surgery). The Ane and Sur groups were treated with an equal volume of saline. The doses of clenbuterol were chosen based on earlier reports [24].

### 2.3. Morris Water Maze

The Morris water maze (MWM) is a hippocampus-dependent spatial navigation and reference memory test for rodents. The MWM was performed as described previously, albeit with minor modifications [25]. Mice underwent daily testing with three trials per day for five consecutive days before surgery and then for 7 days after surgery. Time spent to locate the platform, swimming speed, and distance was recorded by water maze software (Sunny Instruments Co. Ltd., Beijing, China). On postoperative days 3 and 7, a probe test was performed to evaluate memory consolidation; the platform was removed and mice were allowed to swim for 90 s. Time percentage in a specific target quadrant and number of crossings over a previously hidden platform were recorded.

### 2.4. Fear Conditioning Test (FCT)

The FCT was based on a previous study with minor modifications [16]. The test phase of the FCT includes a context test that reflects hippocampal-dependent memory and a tone test that evaluates hippocampal-independent memory. This test consists of a training phase 1 day before surgery and a test phase 3 and 7 days after surgery. Learning and memory abilities were assessed based on the percentage of time that the mice demonstrated “freezing” defined as no movements except for respiration. And freezing time was recorded by Xeye Fcs software (MacroAmbition S&T Development Co. Ltd., Beijing, China).

### 2.5. Cell Culture and Treatment

Rat pheochromocytoma PC12 cells were purchased from the China Infrastructure of Cell Line Resource (Beijing, China) and cultured in Dulbecco's modified Eagle's medium (DMEM) (Gibco; Invitrogen, Waltham, MA, USA) supplemented with 10% horse serum (Invitrogen), 5% fetal bovine serum (Invitrogen), and 100 *μ*g/ml penicillin/streptomycin (Invitrogen) at 37°C with 5% CO_2_. The cells were maintained in culture dishes precoated with 0.5 mg/ml poly-D-lysine (Sigma, St. Louis, MO, USA) to improve cell adherence. The medium was replaced by DMEM containing 1% horse serum and 50 ng/ml nerve growth factor (NGF) (Sigma) to induce neural cell differentiation 24 h after seeding. Half of the NGF medium was refreshed every other day. Five days later, the differentiated PC12 cells (Figure S1b) were treated with LPS *E. coli* O111:B4 (1 *μ*g/ml; Sigma).

### 2.6. Primary Hippocampal Cell Culture

Primary hippocampal neurons were prepared using a previously described method [24]. Brains of neonatal (0–24 h) rats were isolated. The hippocampus was separated and chopped into pieces on an ice bath after careful removal of the meninges. The hippocampus was dissected in Hank's Balanced Salt Solution buffered with Hepes and dissociated with 0.125% (*w*/*v*) trypsin (Invitrogen) digestion for 15 min, then was vigorously mixed in DMEM with 10% (*v*/*v*) fetal bovine serum. Cells were plated on poly-D-lysine precoated dishes at 37°C in 5% CO_2_. After 6 h, cultures were maintained in neurobasal plating media supplemented with B27 Supplement (1 ml/50 ml; Invitrogen), 0.5 mM glutamine solution (Invitrogen), and 100 *μ*g/ml penicillin/streptomycin. Half of the media was replaced every 3 days. Neuronal purity was assessed by western blotting for ionized calcium-binding adaptor molecule 1 (Iba1) and glial fibrillary acidic protein (GFAP) expression. Microtubule-associated protein 2 (MAP-2) staining was also performed (1 : 500; Cell Signaling Technology, Inc., Danvers, MA, USA) (Figure S1a). Primary neurons were used for experiments on days 9–11.

### 2.7. Lentivirus Construction and Transfection

For loss of function experiments using shRNA, lentiviral vector particles containing *Snca*-shRNA (NM_019169.2) were constructed and synthesized by Shan Dong ViGene Co., Ltd., (Jinan, China). The primers for *Snca* were forward: 5′-GTGGCTGCTGCTGAGAAAAC-3′ and reverse: 5′-TCCATGAACGACTCCCTCCT-3′. The viral titer of Lv-shSnca was 1.0 × 10^8^ transducing units (TU)/ml. NGF-differentiated PC12 cells and primary hippocampal neurons were transfected with Lv-shSnca or a control lentivirus (LV-shControl) for 72 h. The expression of SNCA was detected by western blotting (Figure S1c).

### 2.8. Dot Blot

For dot-blotting [26], lysates (1 *μ*g per dot) were spotted onto polyvinylidene difluoride membrane using a narrow-mouth pipette tip and air-dried. After blocking in 5% bovine serum albumin in tris-buffered saline with Tween 20 for 1 h at room temperature, the blot was incubated with a mouse monoclonal antibody against total SNCA (4D6, 1 : 5000) (ab1903; Abcam, Cambridge, UK). After subsequent washes, the blot was incubated with 1 : 2000 anti-rabbit horseradish peroxidase- (HRP-) conjugated secondary antibody (goat anti-mouse immunoglobulin/HRP) and then developed with enhanced chemiluminescence reagent. The relative amount of *α*-syn in samples was quantified by Gel-Pro analyzer software (Rockville, MD, USA).

### 2.9. Western Blots

Cells or hippocampi were lysed in radioimmunoprecipitation assay (RIPA) buffer containing phosphatase and protease inhibitor cocktails (Sigma). The experimental protocol was performed in accordance with a previous study [27]. Antibodies against Bax (50599-2-Ig; Proteintech, Wuhan, China), cytochrome c (Cyt *c*) (10993-1-AP; Proteintech), Bcl-2 (ab32124; Abcam), and cleaved caspase 3 (9661S; Cell Signaling Technology, Inc.) were purchased. *β*-Actin or cytochrome c oxidase (COXIV) antibody (66009-1-lg and 11242-1-AP; Proteintech) was used as internal controls.

### 2.10. Immunohistochemistry

Mice were perfused with cold phosphate-buffered saline (PBS) followed by 4% paraformaldehyde (PFA) under anesthesia. The brain was removed and fixed in 4% PFA at 4°C overnight, according to a previous protocol [26]. After dehydration, clearing, paraffin infiltration, and embedding, the whole hippocampus was sectioned into sections of 6 *μ*m thickness. After antigen retrieval, paraffin tissue sections were blocked using goat serum for 30 min at room temperature. Accumulation of SNCA oligomers was detected by immunohistochemistry using a rabbit polyclonal SNCA oligomer-specific Syn33 antibody (ABN2265; Merck Millipore, Billerica, MA, USA), a mouse monoclonal antibody against total SNCA (4D6, ab1903; Abcam), a rabbit polyclonal COXIV antibody (11242-1-AP; Proteintech), a rabbit monoclonal antibody against p-*α*-syn (Ser129, ab1903; Abcam), and a rabbit monoclonal antibody against Iba-1 (Wako 019-19741; Rosemont, IL, USA). Subsequent incubation was performed using goat anti-rabbit/mouse Alexa Fluor® 594 (ab150116 and ab150080; Abcam) and goat anti-rabbit Alexa Fluor® 488 secondary (ab150077; Abcam) antibodies.

### 2.11. Statistics

Data were analyzed using GraphPad Prism 8.0 software (La Jolla, CA, USA). All data are presented as mean ± standard error of the mean (SEM). Water maze escape latency from three different groups was analyzed by repeated two-way analysis of variance (ANOVA) with Bonferroni post hoc analyses. Other data were analyzed with one-way ANOVA followed by the Bonferroni test. *P* < 0.05 was considered statistically significant.

## 3. Results

### 3.1. Mitochondrial *α*-syn Accumulation and Mitochondrial Dysfunction in the Hippocampus after Anesthesia plus Surgery

**Figure 2:**
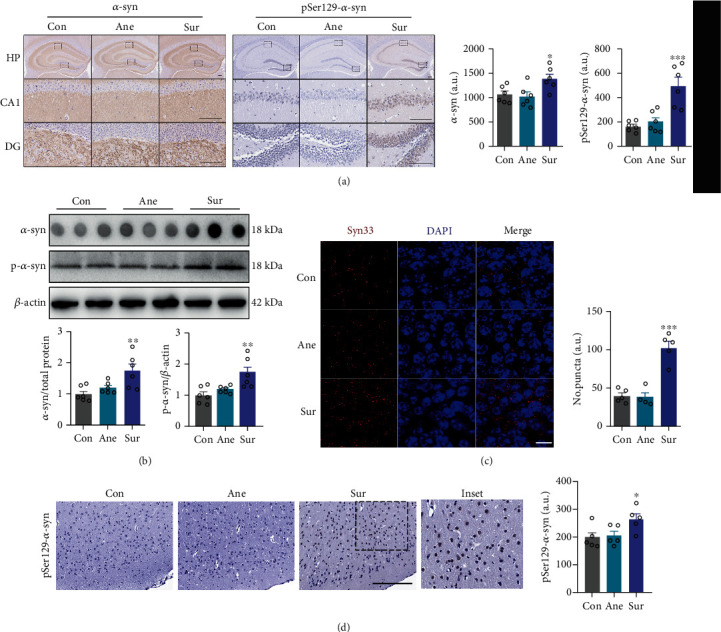
Anesthesia plus surgery increased *α*-syn conformations in mouse brain hippocampus. (a) *α*-syn-positive granules and pSer129-*α*-syn were detected by immunostaining in aged (18-month old) mouse hippocampal CA1 and dentate gyrus (DG) subregions at 24 h after surgery. Signal intensity was quantified (a.u.: arbitrary unit). Scale bars = 100 *μ*m, *n* = 6. (b) Dot blots for total *α*-syn. pSer129-*α*-syn was examined by western blotting. Relative levels of total *α*-syn and pSer129-*α*-syn are shown (*n* = 6). (c) Accumulation of *α*-syn oligomers (red) in aged mouse hippocampus was demonstrated by immunostaining using an *α*-syn oligomer-specific antibody (Syn33). Scale bars = 10 *μ*m, *n* = 5. (d) pSer129-*α*-syn was detected by immunostaining in the frontal cortex of 18-month-old mice at 24 h postsurgery. Signal intensity was quantified (a.u.: arbitrary unit). Scale bars = 100 *μ*m, *n* = 5. Results are presented as mean ± SEM (one-way analysis of variance). ^∗^*P* < 0.05, ^∗∗^*P* < 0.01, ^∗∗∗^*P* < 0.001, Control (Con) or anesthesia-alone (Ane) vs. surgery plus anesthesia (Sur).

Protein aggregation is a common pathological feature of *α*-syn. Therefore, to investigate whether anesthesia plus surgery increases the susceptibility of *α*-syn accumulation in hippocampal soluble lysates of aged mice, both dot blotting and immunohistochemistry were performed. Dot blotting offers no information on the size of the target protein. According to the previous study, it is used to detect total *α*-syn protein expression [26]. We found total soluble *α*-syn was significantly increased in Sur mice compared with those in the Ane and Con groups (Figures 2(a) and 2(b)).

*α*-syn is found in different conformational species, including monomers, oligomers, and fibrils. The formation of fibrils from monomers involves the formation of oligomeric intermediates of different sizes and morphology [28]. Several reports have highlighted *α*-syn oligomers as the species responsible for *α*-syn cytotoxicity [28, 29]. Compared with Con and Ane littermates, accumulation of *α*-syn oligomers was detected in Sur mice by oligomer staining using an *α*-syn oligomer-specific antibody, Syn33. Immunostaining against Syn33 revealed more granular structures deposited in the hippocampus of aged Sur mice (Figure 2(c)). Compared with Sur mice, there were no observable differences in the levels of *α*-syn oligomers in the Con and Ane groups (Figure 2(c)).

Most *α*-syn is unphosphorylated under physiological conditions *in vivo* [30], and phosphorylation of *α*-syn at serine 129 is a marker of pathological *α*-syn and neurotoxicity [31, 32]. Several studies have suggested that phosphorylation of serine 129 may contribute to *α*-syn abnormalities as well as promote oligomer formation. These changes cause aggregation and eventually lead to neuronal cell death [32, 33]. Western blot analysis showed increased p-*α*-syn in the hippocampus of Sur mice compared with Con and Ane mice (Figure 2(b)). This is consistent with the phenomenon of increased expression obtained by immunohistochemistry (Figure 2(a)), and there was no significant difference between Ane and Con mice. Again, we found increased protein expression of p-*α*-syn in the frontal cortex of the Sur group compared to Ane and Con groups (Figure 2(d)).

**Figure 3:**
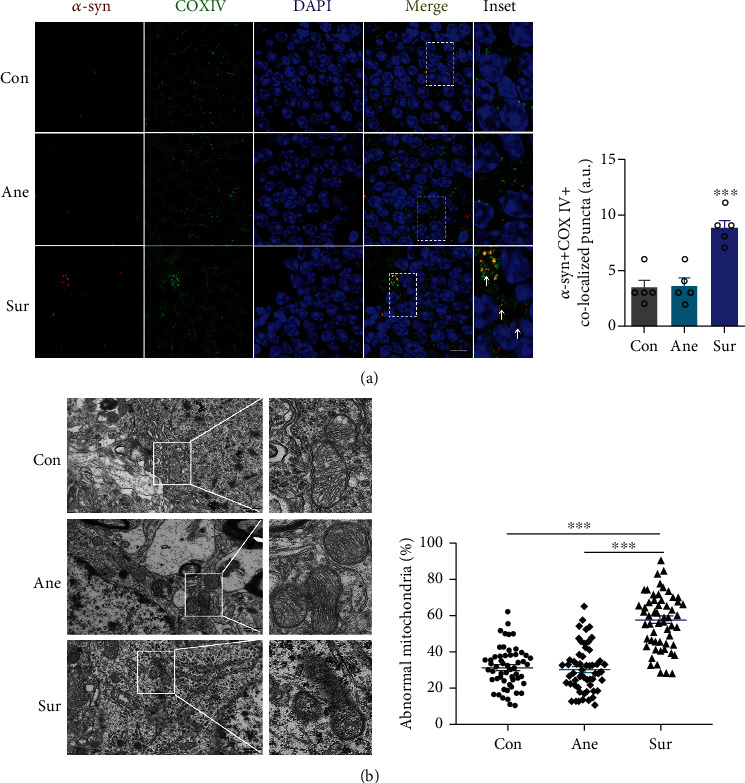
Peripheral surgery increased mitochondrial *α*-syn accumulation and damage. (a) The hippocampus was stained to determine colocalization of *α*-syn (red) and COXIV (an inner mitochondrial membrane (green)). Cell nuclei were stained with DAPI (blue). Arrows point to mitochondrial *α*-syn; scale bars = 10 *μ*m, *n* = 5. (b) Transmission electron microscopy showing mitochondrial morphology in the hippocampus after surgery (*n* = 3 mice per group) and assessment of abnormal cristae (*n* = 57 − 60 cells). Scale bars = 500 nm. Results are presented as mean ± SEM (one-way analysis of variance). ^∗∗∗^*P* < 0.001, control (Con) or anesthesia-alone (Ane) vs. surgery plus anesthesia (Sur).

Multiple lines of evidence support the importance of hippocampal mitochondrial homeostasis in the development of dNCR after anesthesia and surgery, although the precise mechanisms merit additional exploration [5, 8]. We reasoned that mitochondria might play a key role in preventing cognitive impairment after anesthesia and surgery. Thus, we performed further experiments to examine mitochondrial morphology by TEM. An increased number of damaged mitochondria was observed in the Sur group compared with the Con and Ane groups (Figure 3(b)). Detailed examination revealed that Sur mice had many fragmented mitochondria, and the membranes of which were disrupted and without clear cristae (Figure 3(b)). Similar fragmented mitochondria were rarely observed in the Con and Ane mice.

*α*-syn can be located at mitochondria under stress conditions, particularly at the inner mitochondrial membrane (IMM) [20]. Mitochondrial abnormalities have been implicated in *α*-syn toxicity [34, 35]. To examine *α*-syn toxicity in hippocampal mitochondria of aged mice after anesthesia and surgery, we examined colocalization of *α*-syn with COXIV (an IMM protein) by confocal microscopy. Our results showed that mitochondrial *α*-syn was significantly aggregated in the Sur group (Figure 3(a)). Collectively, these data suggest there is greater deposition of *α*-syn granules and mitochondrial *α*-syn in the hippocampus of dNCR mice.

### 3.2. Surgery plus Anesthesia Induced Cognitive Impairment in Aged Mice

Previous work in our laboratory has demonstrated that surgery plus anesthesia, but not anesthesia alone, leads to spatial learning and memory deficits in a mouse model of Alzheimer's disease [25]. Next, we evaluated the influence of anesthesia plus surgery on learning and memory in aged mice using the MWM. There was no significant difference among the three groups in water maze training before surgery, which suggests that all mice shared similar cognitive functions. Mice subjected to anesthesia and surgery displayed higher escape latencies on test days 2 and 3 (Figure 1(b)). On day 4, the impaired performance of the Sur group in escape latency returned to control levels, and there was no significant difference among all groups (Figure 1(b)). Shorter platform site times and fewer times traveled in the target quadrant on probe test day 3, but not day 7, were observed in the Sur group compared with the Con and Ane groups (Figures 1(c) and 1(d)). There was no significant difference in water maze test results between the Con and Ane groups (Figures 1(b)–1(d)). Comparable swimming speeds among groups suggest that the impaired performance in the Sur group was not a result of reduced locomotor ability (Figure 1(e)). Hence, our results suggest that surgery causes cognitive impairment, specifically a deficit in short-term memory retention.

### 3.3. LPS Induced Mitochondrial *α*-syn Accumulation in NGF-Differentiated PC12 Cells and Primary Hippocampal Neurons

Previous studies, including ours, have demonstrated that neuroinflammation plays a key role in the development of dNCR. LPS can trigger a systemic inflammatory response. We and other researchers have used intraperitoneal injection of LPS to simulate anesthesia and surgery, which is an effective animal model for dNCR [27, 36]. Therefore, in this study, we continued to use LPS-induced neurons *in vitro*.

To determine the role of *α*-syn in both NGF-differentiated PC12 cells and rat primary hippocampal neurons, cells treated with LPS were freshly sonicated in cold PBS with protease inhibitors to extract protein. As shown in (Figure S2), LPS (0.5, 1, and 5 *μ*g/ml) treatment for 6 h significantly increased the expression of both *α*-syn and pSer129-*α*-syn in a dose-dependent manner in differentiated PC12 cells (Figure S2a) and rat primary hippocampal neurons (Figure S2b). Mitochondrial and cytosolic proteins were extracted; then, we examined the subcellular localization of *α*-syn. As expected, we observed that LPS markedly increased mitochondrial and cytoplasmic *α*-syn expression in NGF-differentiated PC12 cells (Figure S2c) and primary hippocampal neurons (Figure S2e), as determined by western blotting. MitoTracker Red was used to label mitochondria, and we detected considerably more mitochondrial *α*-syn in LPS stimulated NGF-differentiated PC12 cells (Figure S2d) by immunofluorescence analysis. Similar increases in mitochondrial *α*-syn were observed in LPS-treated rat hippocampal neurons (Figure S2f). These *in vitro* results were consistent with increased accumulation of *α*-syn in the hippocampus of aged mice.

### 3.4. The Mitochondrial Caspase-Dependent Apoptosis Pathway Was Activated after dNCR In Vivo and LPS Exposure In Vitro

To further examine neuronal apoptosis, we used TUNEL staining and western blotting to examine cell apoptosis in the hippocampus. Few TUNEL^+^ cells were detected in the hippocampus from Con and Ane mice. Compared with those groups, the number of TUNEL^+^ cells was significantly increased in Sur mice, with the majority of apoptotic cells being neurons, especially in the dentate gyrus subregion (Figure S3a). Apoptotic stimuli can activate the apoptosis-related proteins, Bax and Bcl-2, to enter mitochondria and induce the release of Cyt *c*. Subsequent activation of caspase 3 represents a key step in the mitochondrion-dependent apoptotic pathway [37]. Here, we found that the Bax/Bcl-2 ratio, Cyt *c*, and caspase 3 cleavage were increased in Sur mice but decreased in Con and Ane mice by western blot analysis (Figure S3b).

We then investigated the apoptosis of NGF-differentiated PC12 and rat primary hippocampal neurons *in vitro*. Selected apoptosis-related proteins were determined by western blotting. Our results showed that the Bax/Bcl-2 ratio increased in differentiated PC12 cells at 24 h after LPS exposure (Figure S3d). To determine whether LPS exerts its apoptotic action via this mitochondrial-dependent signaling pathway, Cyt *c* abundance and cleaved caspase-3 were also measured. The western blotting analysis showed that Cyt *c* expression levels increased in the cytosolic fraction following LPS exposure, whereas they decreased in mitochondria (Figure S3e). Similarly, LPS significantly increased caspase 3 cleavage in differentiated PC12 cells (Figure S3d). These results are consistent with our observations in primary hippocampal neurons (Figure S3g). Next, we performed CCK8 assays to quantitate cell viability. PC12 cells treated with various LPS concentrations (0.5, 1, and 5 *μ*g/ml) for 24 h caused a concentration-dependent decrease in cell viability (Figure S3c). Furthermore, the percentage of TUNEL^+^ cells significantly increased in primary hippocampal neurons in accordance with the LPS concentration gradient (Figure S3f). Our data confirms that the mitochondrial-dependent apoptosis pathway is activated in dNCR mice and LPS-induced neurons.

### 3.5. Treatment with Clenbuterol Attenuated Mitochondrial *α*-syn Accumulation and Improved Cognitive Deficits in dNCR Mice

**Figure 4:**
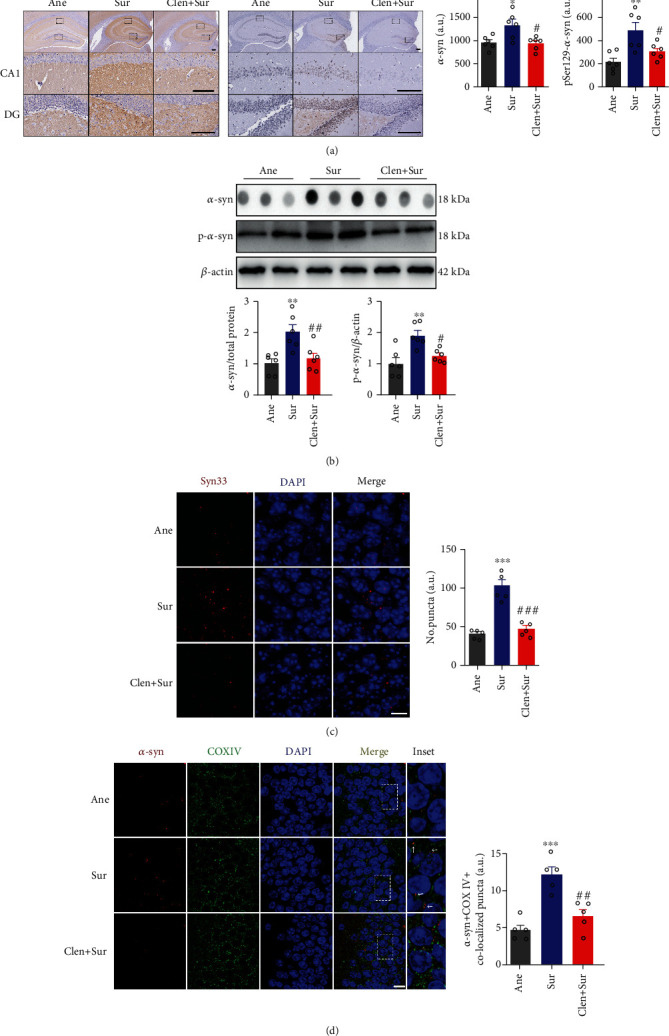
Clenbuterol attenuated mitochondrial *α*-syn accumulation in the hippocampus of delayed neurocognitive recovery mice. (a) Representative photomicrographs and statistical analysis of immunohistochemistry of total *α*-syn and pSer129-*α*-syn in the hippocampal CA1 and dentate gyrus (DG) subregions of delayed neurocognitive recovery mice. Scale bars = 100 *μ*m. (b) Protein levels of total *α*-syn dot blots and pSer129-*α*-syn western blots in the hippocampus; quantification of relative intensities. (c) Immunofluorescence of *α*-syn oligomers (Syn33, red) in mouse hippocampus at 24 h after surgery, with mean fluorescence of Syn33. Scale bars = 10 *μ*m. (d) Immunofluorescence of *α*-syn (red) in mitochondria (green) of the hippocampus. Arrows point to mitochondrial *α*-syn, scale bars = 10 *μ*m; *n* = 5–6/group. Data are expressed as mean ± SEM. ^∗^*P* < 0.05, ^∗∗^*P* < 0.01, ^∗∗∗^*P* < 0.001, anesthesia-alone (Ane) vs. surgery plus anesthesia (Sur); ^#^*P* < 0.05, ^##^*P* < 0.01, ^###^*P* < 0.001, surgery plus anesthesia (Sur) vs. surgery plus clenbuterol (Clen+Sur).

*α*-syn exists in a dynamic equilibrium among various conformations and oligomers, and the propensity for its aggregation may be reversed by reduction in monomeric *α*-syn expression, which results in disaggregation of soluble oligomers [38]. Clenbuterol is a selective beta-2 adrenergic receptor (*β*2AR) agonist, which can be efficiently administered intraperitoneally and cross the blood–brain barrier, and its brain/plasma ratio increases in a dose-dependent manner [24]. *β*2AR agonists promote dopamine neuronal health by reducing SNCA expression (through H3K27 deacetylation) and mitochondrial free radicals [24, 39]. We first determined whether clenbuterol treatment could reduce monomeric *α*-syn and further inhibit *α*-syn aggregation in the hippocampus. Our results showed that surgery-induced total *α*-syn upregulation was abolished by clenbuterol treatment in the Sur+Clen group (Figures 4(a) and 4(b)). A similar reduction of pSer129-*α*-syn levels was observed (Figures 4(a) and 4(b)). Compared with the Sur group, clenbuterol administration markedly decreased the formation of *α*-syn oligomers in the hippocampus (Figure 4(c)). Furthermore, we observed that the levels of mitochondrial *α*-syn in the hippocampus were increased in the Sur group compared with the Ane group, which was reversed by clenbuterol treatment (Figure 4(d)).

**Figure 5:**
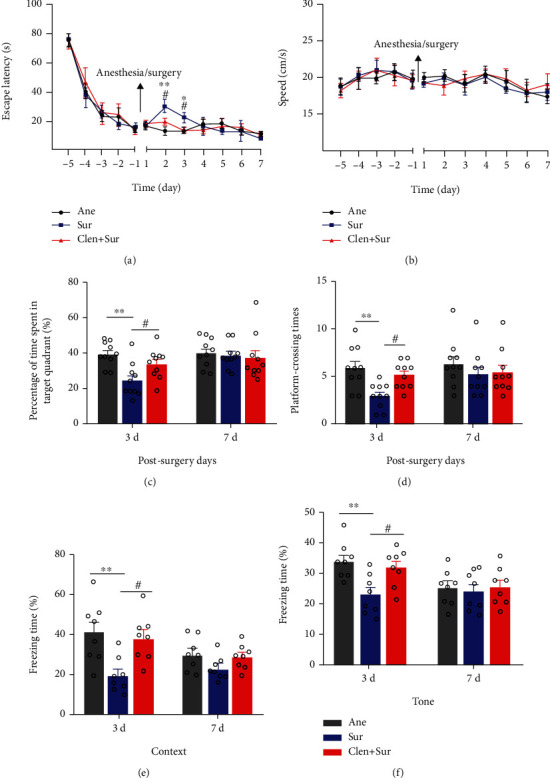
Clenbuterol ameliorated the surgery-induced cognitive impairment in aged mice. (a) Escape latency during the Morris water maze (MWM) navigation test (*n* = 10). (b) The swimming speed in the MWM test. (c) Percentage of target quadrant dwelling time in the MWM test. (d) Platform site crossings during the probe trial. (e) Freezing time in context-related fear conditioning test (*n* = 8). (f) Freezing time in the tone test. Data are expressed as mean ± SEM. ^∗^*P* < 0.05, ^∗∗^*P* < 0.01, anesthesia-alone (Ane) vs. surgery plus anesthesia (Sur); ^#^*P* < 0.05, surgery plus anesthesia (Sur) vs. surgery plus clenbuterol (Clen+Sur).

We also performed the two behavioral assays to examine changes in learning and memory function. In the MWM test, there were no marked differences among the three groups in water maze training before surgery (Figures 5(a) and 5(b)). The Sur group exhibited extended escape latencies on the 2nd and 3rd days after surgery (Figure 5(a)). Further, in the probe test, we observed fewer platform crossings and shorter target quadrant times by mice in the Sur group compared with those in the Ane group (Figures 5(c) and 5(d)). After clenbuterol treatment, the latency to find the platform was shorter on day 3 of training by mice in the Sur+Clen group compared with those in the Sur group. Further, the frequency of platform crossings and time spent in the target quadrant also significantly increased (Figures 5(c) and 5(d)), thereby confirming a beneficial effect of clenbuterol on cognition. No significant difference was observed in swimming speed among groups (Figure 5(b)). In the FCT, the Sur group showed lower freezing time to context (day 3) and tone (day 3) than the Ane group, which was reversed by Clen pretreatment (Figures 5(e) and 5(f)). However, there was no significant difference in freezing time among the three groups on day 7 after surgery (Figures 5(e) and 5(f)).

Taken together, these findings suggest that clenbuterol treatment abrogated surgery-induced *α*-syn accumulation and subcellular localization of *α*-syn in mitochondria, and importantly, also improved cognitive deficit in dNCR mice.

### 3.6. Blocking *α*-syn Mitigated Mitochondrial-Dependent Apoptosis and Microglia Activation of Hippocampal Neurons in dNCR Mice

**Figure 6:**
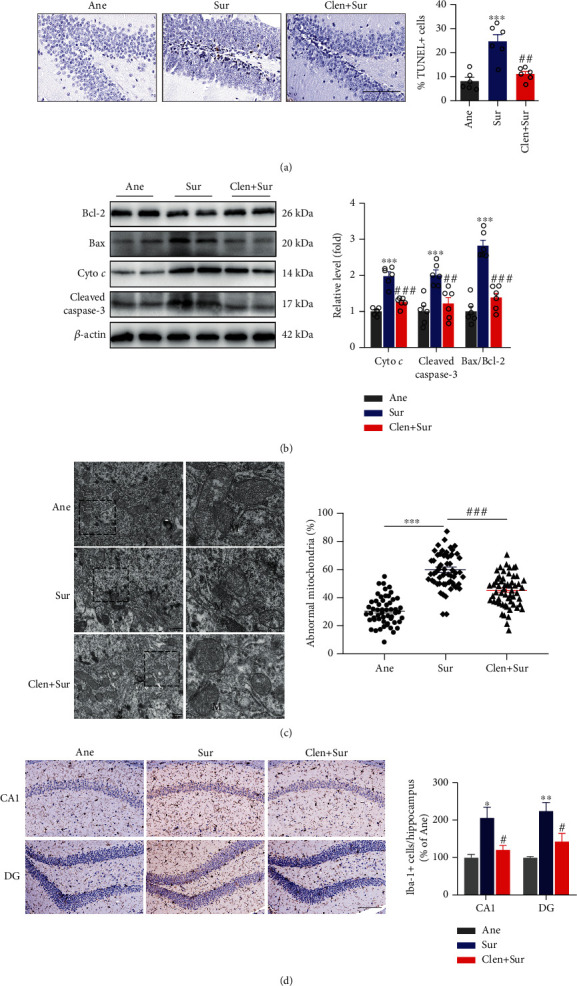
Blocking *α*-syn attenuated mitochondrial-dependent apoptosis of hippocampal neurons in delayed neurocognitive recovery mice. (a) TUNEL staining of the hippocampus at 24 h after surgery pretreated with clenbuterol and quantitation of TUNEL staining. (b) Western blot analysis of cytochrome c (Cyt *c*), cleaved caspase 3, Bcl-2, and Bax in the hippocampus following surgery, with or without clenbuterol treatment. Quantification of the data is shown. (c) Mitochondrial morphology in the hippocampus, *n* = 3 mice per group and quantification of abnormal cristae (*n* = 57 − 60 cells). (d) Representative photomicrographs of the immunohistochemical analysis of microglia (Iba-1-positive cells) in the hippocampal CA1 and DG subregions of mice and their statistical analysis (scale bars: 100 *μ*m; *n* = 3). Data are expressed as mean ± SEM (one-way analysis of variance, *n* = 5/group). ^∗^*P* < 0.05, ^∗∗^*P* < 0.01, ^∗∗∗^*P* < 0.001, anesthesia-alone (Ane) vs. surgery plus anesthesia (Sur); ^#^*P* < 0.05, ^##^*P* < 0.01, ^###^*P* < 0.001, surgery plus anesthesia (Sur) vs. surgery plus clenbuterol **(**Clen+Sur).

Since accumulated *α*-syn-mediated toxicity to neurons is associated with mitochondrial dysfunction, we determined whether inhibition of *α*-syn could maintain neuronal mitochondrial homeostasis in dNCR mice *in vivo*. We first performed TEM on hippocampal sections from dNCR mice. Ultrastructural examination of the hippocampus from Con mice showed intact normal mitochondria. Importantly, 60% of the mitochondrial degradation and vacuolization were evident following anesthesia plus surgery treatment (Sur group) (Figure 6(c)), clenbuterol treatment effectively bated the surgery-induced mitochondrial damage. Microglia activation and neuroinflammation are involved in dNCR after surgery, and we detected the effects of clenbuterol on Sur group mice microglial activation by immunohistochemistry. Pretreatment of clenbuterol significantly attenuated the operation-induced activation of microglia (Iba-1) in the CA1 and DG subregions of the hippocampus. (Figure 6(d)). Thus, this data indicated that microglial activation may be involved in the pathogenesis of SNCA-mediated dNCR in aged mice.

To examine the effect of clenbuterol treatment on neural apoptosis in the hippocampus, TUNEL staining was performed to detect apoptotic cells after surgery. Cellular apoptosis following surgery was indicated by many TUNEL^+^ cells throughout the dentate gyrus region compared with sevoflurane anesthesia alone (Ane group) (Figure 6(a)). Perioperative administration of clenbuterol consistently reduced TUNEL^+^ cells following laparotomy in the dentate gyrus (Sur+Clen group). We also found significantly high levels of Bax/Bcl-2, Cyt *c*, and cleaved caspase 3 in the hippocampus of postsurgical mice (Sur group) compared with the Ane group. After perioperative administration of clenbuterol, significant reductions in the Bax/Bcl-2 ratio, Cyt *c* release, and cleaved caspase 3 were observed (Figure 6(b)). This is consistent with limited neuronal apoptosis shown by TUNEL staining, suggesting clenbuterol prevents surgery-induced apoptosis in the hippocampus. These results provide insight into the molecular mechanism of the antiapoptotic action of *α*-syn inhibition *in vivo*, which acts in a caspase-dependent apoptotic pathway.

### 3.7. Specific Deletion of *α*-syn Improved Mitochondrial Dysfunction in Neuronal Cell Lines

**Figure 7:**
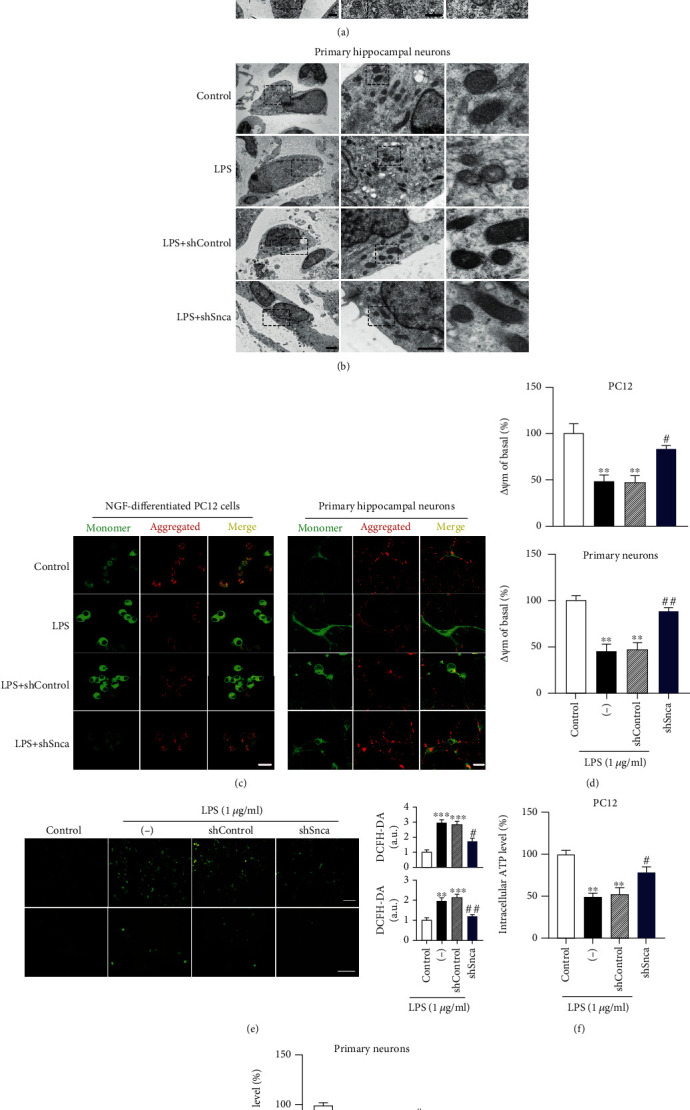
*α*-syn inhibition protected mitochondria against lipopolysaccharide-induced dysfunction in neuronal cells. *α*-syn inhibition in both cell models was performed with transfected Lv-shSnca for 48 h following lipopolysaccharide **(**LPS) exposure. (a and b) Transmission electron microscopy was used to examine mitochondrial morphology in nerve growth factor- (NGF-) differentiated PC12 cells (a) rat primary hippocampal neurons (b). Scale bar = 500 nm. (c) Mitochondrial membrane potential (*ΔΨ*m) was assessed using JC-1 (10 *μ*g/mL) in both neuronal cells. Scale bars = 20 *μ*m. (d) Fluorescent intensity was analyzed. (e) Total cellular reactive oxygen species production detected with DCFH-DA in NGF-differentiated PC12 cells (upper) and rat primary hippocampal neurons (lower); scale bars = 50 *μ*m. ATP generation was assessed in NGF-differentiated PC12 cells (f) and rat primary hippocampal neurons (g) using a luminometric assay. Data are expressed as mean ± SEM (one-way analysis of variance, *n* = 3). ^∗∗^*P* < 0.01, ^∗∗∗^*P* < 0.001, control (Con) vs. LPS or LPS+Lv-shControl; ^#^*P* < 0.05, ^##^*P* < 0.01, LPS+Lv-shControl vs. LPS+Lv-shSnca.

Based on the observation that *α*-syn accumulation impaired mitochondrial morphology, we determined whether mitochondrial function was also impaired in a cell model. We used a complementary genetic approach to specifically reduce *Snca* function by transfection with Lv-shSnca. First, we transfected cells with shSnca, which achieved approximately 70–80% of *Snca* knockdown efficiency (Figure S1c). Then, we examined the mitochondrial morphology by TEM. LPS with or without Lv-shControl induced cells that exhibited fragmented mitochondria with vacuoles, and the degree of mitochondrial injury was significantly reversed in Lv-shSnca transfected PC12 and primary hippocampal neurons incubated with LPS (Figures 7(a) and 7(b)).

Next, we measured mitochondrial membrane potential (*ΔΨ*m), which is established by the electrochemical gradient from redox reactions generated by the mitochondrial electron transport chain. A decrease in *ΔΨ*m is an early indicator of mitochondrial dysfunction and plays an essential role in cell apoptosis. Using JC-1 staining, we quantified the red/green ratio of JC-1 staining in both differentiated PC12 cells and primary hippocampal neurons. Cells treated with LPS for 12 h induced a loss or collapse of *ΔΨ*m, while *α*-syn inhibition by Lv-shSnca transfection completely prevented this deficit in both NGF-differentiated PC12 and rat primary hippocampal neurons (Figures 7(c) and 7(d)). No significant difference in *ΔΨ*m was found between LPS and LPS+Lv-shControl (Figures 7(c) and 7(d)).

We further quantified the DCFH-DA signal to detect cellular reactive oxygen species (ROS) levels, an inevitable phenomenon of impaired mitochondrial function. We detected an increase in ROS production in LPS-treated cultures with or without Lv-shControl, and SNCA knockdown in two types of cells can be reversed (Figure 7(e)). Consistent with its inhibitory effect on ROS production, LPS decreased ATP levels, an adverse effect that was blunted by *α*-syn inhibition (Figures 7(f) and 7(g)). Altogether, the ablation of *α*-syn accumulation may protect neurons from LPS-induced mitochondrial dysfunction *in vitro*.

### 3.8. LPS-Induced Mitochondrial-Dependent Apoptosis in Neurons Can Be Modulated by *α*-syn Inhibition

**Figure 8:**
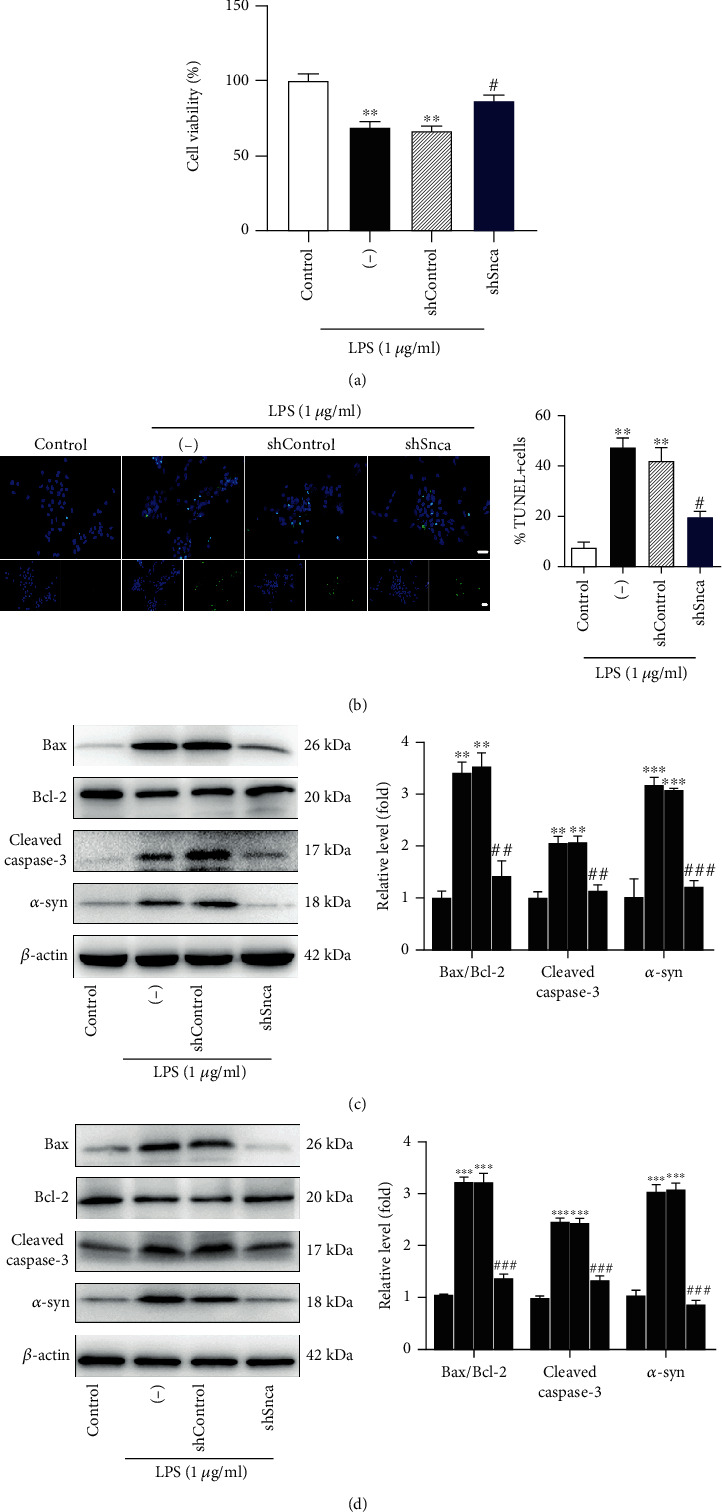
*α*-syn inhibition alleviated lipopolysaccharide induced mitochondrial-dependent apoptosis in neurons. (a) Cell viability was assessed by CCK8 assay in nerve growth factor- (NGF-) differentiated PC12 cells. (b) Apoptosis was detected by TUNEL staining. (c and d) Western blot analysis confirming the effect of *α*-syn knockdown on cleaved caspase 3, Bcl-2, and Bax protein expression in lipopolysaccharide- (LPS-) induced NGF-differentiated PC12 cells (c) and rat primary hippocampal neurons (d). Data are shown as mean ± SEM and analyzed with one-way ANOVA followed by Bonferroni post hoc test, *n* = 3. ^∗∗^*P* < 0.01, ^∗∗∗^*P* < 0.001, control (Con) vs. LPS or LPS+Lv-shControl; ^#^*P* < 0.05, ^##^*P* < 0.01, ^###^*P* < 0.001, LPS+Lv-shControl vs. LPS+Lv-shSnca.

To determine the function of *α*-syn inhibition on neuronal apoptosis induced by LPS, we transfected cells with Lv-shSnca. Like our *in vivo* results, LPS treatment increased the number of TUNEL^+^ nuclei in primary hippocampal neurons. The number of apoptotic nuclei was significantly decreased in Lv-shSnca-transfected neurons following LPS exposure (Figure 8(b)). Apoptosis rate was not significantly different between the LPS and LPS+Lv-shControl groups (Figure 8(b)). As expected, the same phenomenon was also detected in differentiated PC12 cells (Figure S1d). The CCK8 assay in Lv-shSnca+LPS cells also showed higher cell viability compared with LPS-treated differentiated PC12 cells (Figure 8(a)). To further investigate the cellular basis of the apoptotic response observed in neurons, expressions of several apoptosis-related proteins were determined by western blotting. We found that higher expression of the Bax/Bcl-2 ratio was reversed by cotreatment with Lv-shSnca in differentiated PC12 cells (Figure 8(c)) and rat primary hippocampal neurons (Figure 8(d)) compared with LPS treatment with or without Lv-shControl. Similarly, treatment with Lv-shSnca inhibited upregulation of cleaved caspase 3 expression in both cells (Figures 8(c) and 8(d)). These results indicate that inhibition of *α*-syn accumulation could blunt LPS-induced cell apoptosis of neurons.

## 4. Discussion

**Figure 9:**
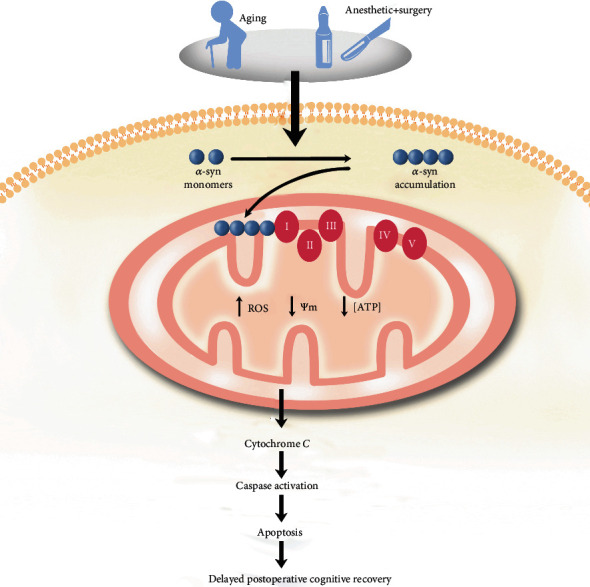
Schematic illustration of the possible toxic mechanism of mitochondrial *α*-syn accumulation in aged dNCR mice.

To support normal cerebral function, the brain has a high energy demand in the form of ATP, which is supplied by mitochondria. There is significant evidence supporting the concept that maintaining mitochondrial homeostasis in neuronal cells is essential for the prevention and treatment of dNCR [5, 7, 40]. The objective of our current study was to identify the specific role of mitochondrial *α*-syn accumulation on neurotoxicity and cognition in aged mice following sevoflurane anesthesia plus surgery and also in cultured neurons exposed to LPS (Figure 9).

*α*-syn is a nonamyloid *β* component of amyloid plaques that were originally found in Alzheimer's disease brains [41] and has since been implicated in the pathogenesis of different forms of Parkinson's disease and dementia with Lewy bodies [42]. Increasing evidence has shown that *α*-syn plays an important role in cognitive function. *α*-syn is primarily localized in axons and presynaptic terminals of neurons in brain regions involved in memory and emotion. Expression of *α*-syn in cerebrospinal fluid is correlated with the speed of cognitive decline in Alzheimer's disease and mild cognitive impairment [43]. *α*-syn oligomers cause memory impairment and oppose long-term potentiation (LTP) in mice by inhibiting transcriptional activity of the cAMP response element-binding protein [44]. Our previous study suggested that *α*-syn accumulation promoted the endocytosis of the NMDA receptor, a key receptor functionally related to memory, and reduced the surface NMDA receptor [45]. Moreover, *α*-syn can also impair the NMDA receptor-mediated LTP, an enhanced synaptic activity, which is widely accepted to be related to memory formation [46]. Meanwhile, our preliminary studies [15, 16] and others [17, 18] have demonstrated that *α*-syn may also be associated with surgery-induced cognitive impairment. However, the exact association between *α*-syn accumulation and the mechanism of dNCR is not fully understood. This current study provides the first evidence that anesthesia plus surgery specifically contributes to mitochondrial *α*-syn accumulation in the hippocampus of aged mice and LPS-induced NGF-differentiated PC12 cells and primary hippocampal neurons.

Further, *α*-syn inhibition following clenbuterol treatment also abolished microglia activation and cognitive impairment induced by anesthesia plus surgery. These data indicate that anesthesia plus surgery can induce a SNCA-dependent cognitive impairment in elderly mice.

Mitochondrial homeostasis plays a critical role in maintaining neuronal survival. Therefore, we and other researchers have provided insight into how suboptimal mitochondria may contribute to cognitive decline and neurodegenerative disorders [47, 48]. Mitochondrial dysfunction and impaired integrity may be pervasive in dNCR, although the exact mechanism is ambiguous. In our present study, through both *in vivo* and *in vitro* experiments, we show that *α*-syn accumulation can enhance mitochondrial dysfunction and morphological defects and may contribute to memory decline. Specific *α*-syn deficiency may have a protective effect on mitochondrial dysfunction in multiple LPS-induced cell models and dNCR mice. Although we only detected mitochondrial shape changes, there is an established association between mitochondrial function and mitochondrial shape, with dysfunctional mitochondria typically exhibiting a smaller more fragmented morphology [49].

A relationship between intracellular *α*-syn accumulation, mitochondrial dysfunction, and bioenergetic impairment has previously been reported in Parkinson's disease. Accumulation of *α*-syn in mitochondria may greatly inhibit complex I activity, leading to reduced ATP production, enhanced ROS generation, and accelerated neurodegeneration in dopaminergic neuronal cultures [20]. *α*-syn incubated with isolated rat brain mitochondria led to a dose-dependent loss of *Δψ*m, mitochondrial potential transition pore opening, and decreased phosphorylation capacity by interacting with the voltage-dependent anion channel (VDAC) and adenine nucleotide translocator [50]. Consistent with other studies, we previously reported that *α*-syn in mitochondria is differentially expressed in different brain regions. In particular, *α*-syn is highly expressed in mitochondria in the olfactory bulb, hippocampus, and striatum, where cytosolic *α*-syn was also plentiful [51]. Incubation of *α*-syn with mitochondria caused dose-dependent transport of *α*-syn to mitochondria and a dose-dependent inhibitory effect on complex I activity of the mitochondrial respiratory chain [51]. These studies show that mitochondrial dysfunction is a significant indicator in the early phase of cellular injury induced by *α*-syn. Indeed, *α*-syn knockdown triggered mitochondrial elongation, with elongated structures being more efficient in ATP generation [52, 53]. Rapid morphological adaptations are important for many cellular processes such as apoptosis and mitochondrial quality control. We had previously shown that *α*-syn mainly localized in the IMM fraction [51]; therefore, we detected colocalization of *α*-syn and COXIV (an IMM marker) to determine mitochondrial levels of *α*-syn. Here, we also show that the levels of mitochondrial *α*-syn may be a potential factor affecting mitochondrial function and predisposing certain neurons to apoptosis. We observed mitochondrial dysfunction in both cell models, including less ATP production, more ROS generation, and loss of *ΔΨ*m. These indicators may be due to the inhibition of complex I activity and loss of VDAC1 induced by mitochondrial *α*-syn aggregation. Nevertheless, the specific mechanisms still need further research.

Alternatively, dysfunctional mitochondria can lead to dysregulation of calcium homeostasis and raised ROS, which can induce increased *α*-syn expression and formation of *α*-syn inclusions [54]. As calcium binding to *α*-syn is an important trigger of *α*-syn aggregation, mitochondrial dysfunction will promote aggregation and disease progression [55]. Increased ROS also contributes to the formation of *α*-syn aggregates and neuronal loss [56]. Therefore, the relationship between *α*-syn and mitochondrial homeostasis is complex and warrants further investigation.

Mitochondrial dysfunction is an important factor associated with apoptosis. The intrinsic mitochondria-dependent pathway is regulated by the Bcl-2 family and consists of the antiapoptotic protein, Bcl-2, and proapoptotic protein, Bax. The imbalance of Bax/Bcl-2 may lead to a decrease of *ΔΨ*m and Cyt *c* release [57]. Cyt *c* is released from mitochondria into the cytosol upon apoptotic stimulation, leading to the formation of the apoptosome and activation of downstream caspase 3, and eventually apoptosis [57]. In the present study, *Snca* knockdown upregulated Bcl-2 and downregulated Bax. Subsequently, decreased release of Cyt *c* and inhibition of caspase 3 activation may ultimately correct the execution of apoptosis in the hippocampus of dNCR mice and neurons exposed to LPS. To summarize, anesthesia plus surgery activated the mitochondrial caspase-dependent apoptotic pathway that is mediated by *Snca* activation.

Our study has certain limitations. To mimic the injury caused by surgical trauma, we used LPS treatment to induce mitochondrial damage and increase *α*-syn in our *in vitro* experiments. LPS can induce neuroinflammation, which also impairs memory. Although the utility of this *in vitro* model has been demonstrated in previous reports [27], it only provides limited *in vivo* relevance regarding the actual process of dNCR pathogenesis. Additionally, although we observed an effect of *α*-syn accumulation on mitochondrial dysfunction, the specific molecular mechanism is still not clear. Our next step is to determine downstream molecular targets of *α*-syn that are responsible for the mitochondrial dysfunction.

In conclusion, our study shows that mitochondrial *α*-syn accumulation after anesthesia and surgical trauma significantly disrupts mitochondrial homeostasis and promotes mitochondria-dependent apoptosis. This ultimately leads to hippocampal-dependent memory in dNCR mice. Administration of the *β*2AR agonist, clenbuterol, may provide cognitive protection by decreasing intracellular *α*-syn accumulation in our mouse model of dNCR. While these findings warrant further investigation to identify interacting proteins of *α*-syn in mitochondria and the pathways implicated in *α*-syn neurotoxicity, we believe that targeting toxic *α*-syn is a promising therapeutic target for dNCR.

## Data Availability

The data used to support the findings of this study are included within the article.

## Supplementary Materials

Supplementary Materials Supplementary materials include some experimental methods and other results. Supplemental Figure 1: identification of hippocampal neurons and detection of lentivirus transfection efficiency. Supplemental Figure 2: lipopolysaccharide induced mitochondrial *α*-syn expression in nerve growth factor-differentiated PC12 cells and primary hippocampal neurons. Supplemental Figure 3: activation of the mitochondrial caspase-dependent apoptosis pathway after delayed neurocognitive recovery *in vivo* and lipopolysaccharide exposure *in vitro*.
