# Supplementary material for: Inhibition of α-Synuclein Accumulation Improves Neuronal Apoptosis and Delayed Postoperative Cognitive Recovery in Aged Mice
Source: Oxid Med Cell Longev. 2021 May 28;2021:5572899. doi: 10.1155/2021/5572899 (PMC8181110; doi:10.1155/2021/5572899)
Supplement: Supplementary Materials — include some experimental methods and other results. Supplemental Figure 1: identification of hippocampal neurons and detection of lentivirus transfection efficiency. Supplemental Figure 2: lipopolysaccharide induced mitochondrial α-syn expression in nerve growth factor-differentiated PC12 cells and primary hippocampal neurons. Supplemental Figure 3: activation of the mitochondrial caspase-dependent apoptosis pathway after delayed neurocognitive recovery in vivo and lipopolysaccharide exposure in vitro. [file 5572899.f1.zip › Supplementary information (1).docx]

**Supplementary information**

**Methods**

**Cell viability assay.** The Cell Counting Kit-8 (CCK8; Dojindo Laboratories, Tokyo, Japan) was used to determine the cell viability of PC12 cells. For the CCK8 assay, 10 μl CCK8 was added to each well of a 96-well plate, which was incubated at 37°C for 2 h. The absorbance at 450 nm was measured using a microplate reader (Thermo Scientific, Waltham, MA, USA).

**Measurement of ATP Synthesis.** ATP content was measured by luminometric assay, based on luciferin-luciferase reactions (Beyotime, Shanghai, China). Brieﬂy, cells were lysed in a buﬀer that contained10 mmol/L Tris and 0.05% Triton X-100. After, 100 µL of this lysate was added, and the luciferin-luciferase assay mixture was transferred to a white microplate. The results were measured on a multifunctional microplate reader (Thermo Scientific).

**ROS measurement.** The levels of intracellular ROS were measured using DCFH-DA (Sigma). Brieﬂy, after LPS treatment for 12 hours, cells were incubated with 10 *μ*M DCFH-DA working solution for 30 minutes at 37˚C in the dark. Fluorescence signals of ROS were detected with excitation and emission wavelengths at 488/525 nm under a TCS SP8 fluorescence microscope (Mannheim, Germany).

**MMP (∆ψm) assay.** Mitochondrial membrane potential (ΔΨm) was assessed by using the JC-1 probe (Sigma). According to suppliers’ recommendation, cells were incubated with 10 μg/mL JC-1 for 30 minutes after 12 h LPS exposure at 37°C in the dark and ultimately observed under LSCM. Meanwhile, the red/green ratio semi-quantitatively was analyzed by ImageJ software (NIH, Bethesda, MD, USA).

**TUNEL assay.** Terminal deoxynucleotidyl transferase (TDT)- mediated dUTP nick end labeling (TUNEL) assay was used to analyze apoptosis, according to the manufacturer’s instructions using In Situ Apoptosis Detection Kit, POD (Roche Basel, Switzerland). Cells with brown nuclei were considered TUNEL-positive. Finally, images were taken using an Olympus BX5 imaging system (Olympus America, Melville, NY, USA) at ×400 magnification. Additionally, a TUNEL assay was performed to assess apoptotic cell death of PC12 or primary hippocampal neurons using In Situ Apoptosis Detection Kit, Fluorescein (Roche). The nuclei were stained with DAPI.

**Immunocytochemistry.** Cells, which were stained with Mito Tracker Red to visualize mitochondria, were plated on confocal and fixed in a mixture of 4% PFA for 15 min at RT. Incubation in 0.3% Triton-X for 5 min permeabilized the cell membrane. Afterward, cells were blocked with 5% BSA in PBS for 45 min before incubated with antibodies against α-syn (#4179; Cell Signaling Technology, Inc.) at 1:100 dilutions in dilution buffer containing 3% BSA overnight at 4°C. After subsequent washes, cells were further incubated with secondary antibody conjugated with Alexa Fluor® 488 (ab150077; Abcam) at 1:500 dilutions for 30 min at RT protected from light. Following PBS washes, cells were stained with DAPI (Sigma) in the dark for 5 min, and then mounted in a mixture of glycerol and PBS (1:1) and observed via LSCM (TCS SP8, Leica).

**Transmission Electron Microscopy (TEM) analysis.** The hippocampal tissue was cut into 2 mm pieces, fixed with 3% glutaraldehyde at 4°C overnight, post-fixed with 1% OsO_4_, 0.8% potassium ferricyanide, and 5mM CaCl2 in 0.1M cacodylate buffer, then dehydrated in acetone and embedded in epoxy resin. Ultrathin sections (100 nm) were observed with a Zeiss transmission electron microscope (JEM-1400, Electron Co., Japan). To quantify the number of mitochondrial abnormalities, extensive image analysis was performed per mouse.

**Mitochondria and cytosol fractionation.** PC12 cells or primary neurons were lysed in a high-efficiency, mitochondrial separation buffer (Beyotime, Shanghai, China) using a homogenizer. Homogenates were centrifuged at 600 × g for 10 min at 4°C to remove the nuclei. After that, the supernatants were collected and centrifuged at 11000 × g for 10 in at 4°C, supernatants were collected as the cytosolic fraction, and the pellet was resuspended into the mitochondrial lysis buffer.

**Supplementary Figure Legends**

**Supplementary Figure 1. Figure S1 a** Primary hippocampal neurons were cultured. 20 μg, 40 μg, and 60 μg protein per lane were used to detect Iba1 and GFAP expression at 10 d to assess the primary neuronal purity. Primary hippocampal neurons were also stained by MAP-2 (green) and DAPI (blue) and confirmed that more than 90 % of MAP-2-positive cells were neurons at 10 d. **b** The morphology after NGF- differentiated PC12 cells 5 days. **c** Primary hippocampal neurons and NGF- differentiated PC12 cells were transfected with Snca shRNA Lentiviral Particles or control lentivirus (LV-shControl) for 72 hours, and the sh-RNA efficiency was detected by western blot. **d** TUNEL staining of the NGF-differentiated PC12 cells and quantitation of TUNEL staining. Data are expressed as mean ±SEM (one-way analysis of variance, n=3). **p<0.01, Con vs. LPS or LPS+shControl; ^#^p<0.05, LPS+shControl vs. LPS+shSnca.

**Figure S2**. Lipopolysaccharide induced mitochondrial α-syn expression in nerve growth factor-differentiated PC12 cells and primary hippocampal neurons. **a** Nerve growth factor (NGF)-differentiated PC12 cells and **b** rat primary hippocampal neurons were stimulated with the indicated concentrations of lipopolysaccharide (LPS) (0.5, 1, and 5 μg/ml) for 6 h, followed by western blotting of α-syn and p-α-syn expression. Data are expressed as mean ± SEM (one-way analysis of variance). **c** Mitochondrial and cytoplasmic proteins were isolated from LPS-induced NGF-differentiated PC12 cells and examined by western blotting. COXIV was used as a mitochondrial protein loading control. β-actin was used as a cytoplasmic protein loading control (Student’s *t*-test). **d** Subcellular localization of α-syn was determined by immunofluorescence analysis with α-syn, MitoTracker Red, and DAPI staining in LPS-treated NGF-differentiated PC12 cells. **e** Mitochondrial and cytoplasmic α-syn were measured by western blotting in rat primary hippocampal neurons (Student’s *t*-test). **f** Mitochondrial α-syn was visualized by immunofluorescence in rat primary hippocampal neurons. Scale bars = 20 μm, *n* = 3. **P* < 0.05; ***P* < 0.01; ****P* < 0.001; ^#^*P* < 0.05; ^##^*P* < 0.01; ^###^*P* < 0.001.

**Figure S3**. Activation of the mitochondrial caspase-dependent apoptosis pathway after delayed neurocognitive recovery *in vivo* and lipopolysaccharide exposure *in vitro*. **a** Quantification of TUNEL staining detected DNA fragmentation in the hippocampus of delayed neurocognitive recovery mice at 24 h after surgery (*n* = 5). **b** Western blotting of hippocampal tissue 24 h following surgery. Quantitative analysis of relative band intensity showing protein levels of apoptotic proteins, including cytochrome c (Cyt *c*), cleaved caspase 3, Bcl-2, and Bax. **c** Nerve growth factor (NGF)-differentiated PC12 cells were cultured with the indicated concentrations of lipopolysaccharide (LPS) for 24 h. Cell viability was determined by CCK8. **d** NGF-differentiated PC12 cells and **g** rat primary hippocampal neurons were exposed to different concentrations of LPS for 24 h. Cell lysates were subjected to western blotting to measure Bax, Bcl-2, and cleaved caspase 3, with quantitative analysis of relative band intensity. **e** NGF-differentiated PC12 cells were treated with LPS. Release of Cyt *c* from mitochondria into the cytoplasm was determined by western blotting and quantification of Cyt *c* in the cytoplasm and mitochondria. COXIV and β-actin were used as loading controls for the mitochondrial and cytoplasmic fractions, respectively. **f** Apoptosis in rat primary hippocampal neurons was detected and quantified using a TUNEL kit at 24 h after LPS exposure. Scale bars = 20 μm. Data are expressed as mean ± SEM (one-way analysis of variance, *n* = 3) **P* < 0.05; ***P* < 0.01; ****P* < 0.001.
