# Supplementary figures and images for: Inhibition of α-Synuclein Accumulation Improves Neuronal Apoptosis and Delayed Postoperative Cognitive Recovery in Aged Mice
Source: Oxid Med Cell Longev. 2021 May 28;2021:5572899. doi: 10.1155/2021/5572899 (PMC8181110; doi:10.1155/2021/5572899)

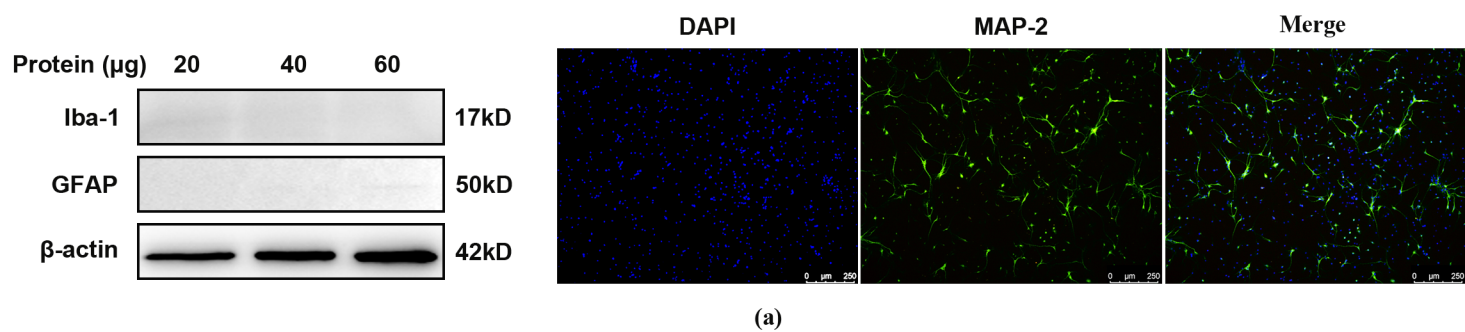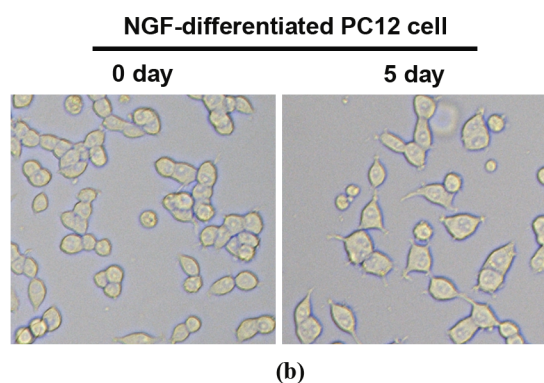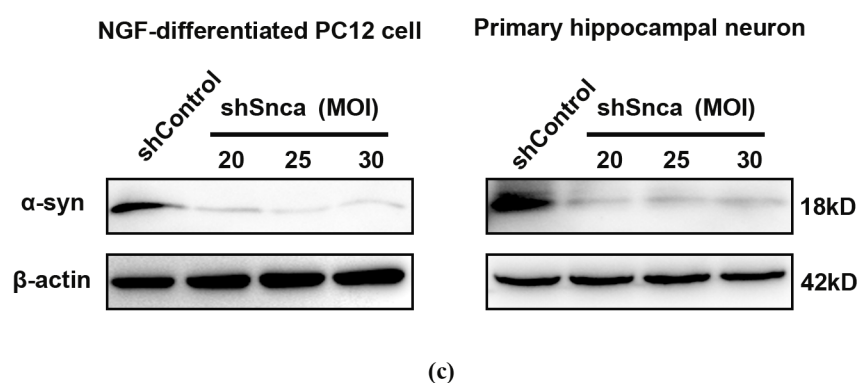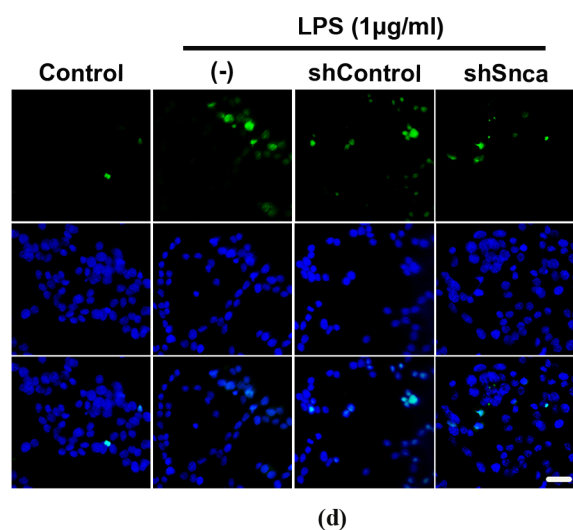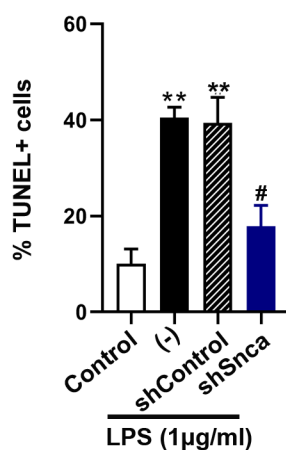

Supplement: Supplementary Materials — include some experimental methods and other results. Supplemental Figure 1: identification of hippocampal neurons and detection of lentivirus transfection efficiency. Supplemental Figure 2: lipopolysaccharide induced mitochondrial α-syn expression in nerve growth factor-differentiated PC12 cells and primary hippocampal neurons. Supplemental Figure 3: activation of the mitochondrial caspase-dependent apoptosis pathway after delayed neurocognitive recovery in vivo and lipopolysaccharide exposure in vitro. [file 5572899.f1.zip › Supplementary Figure 1.pdf]

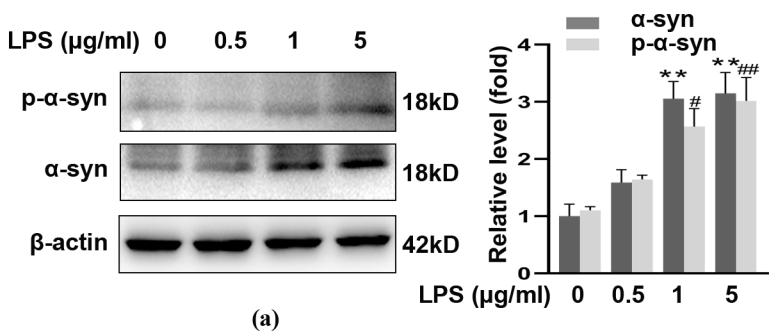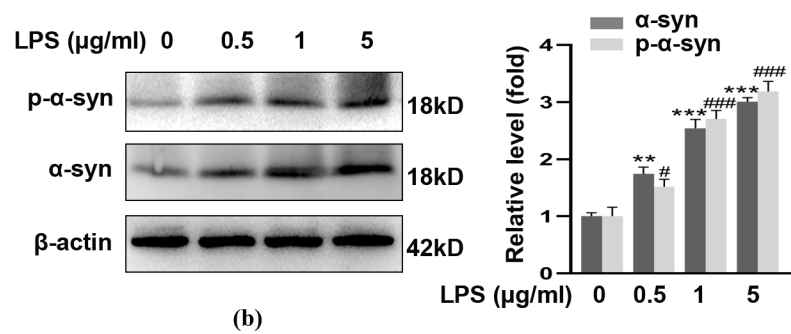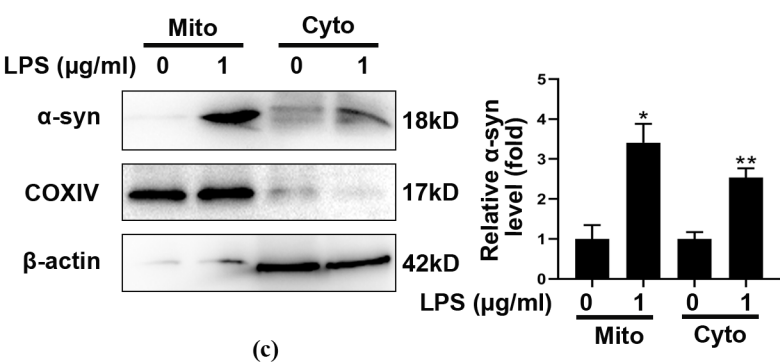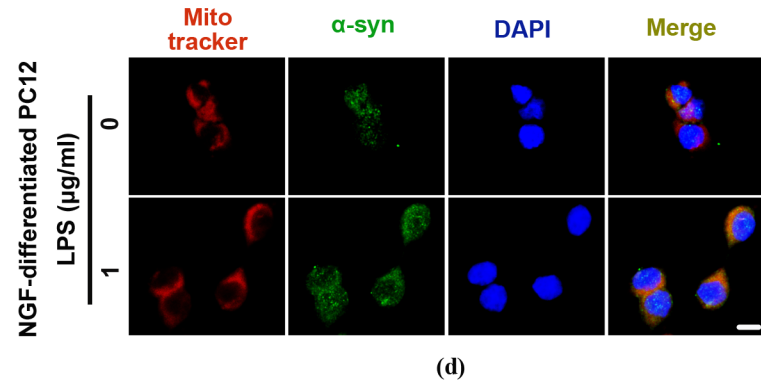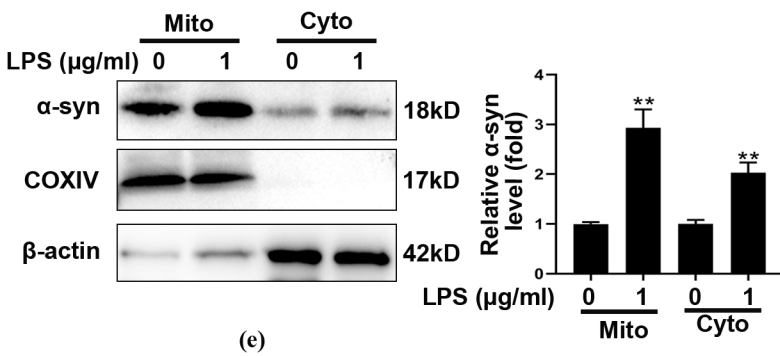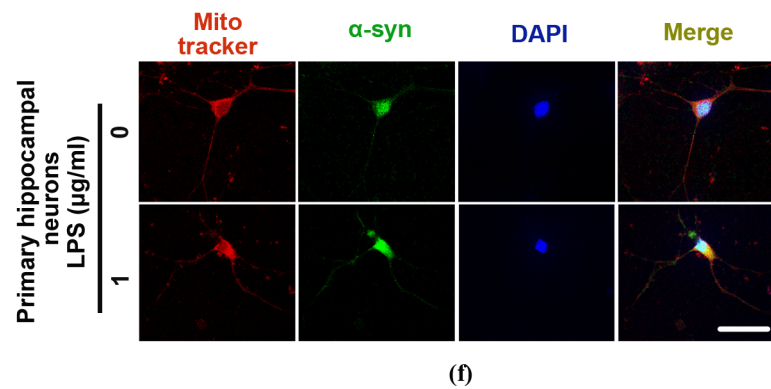

Supplement: Supplementary Materials — include some experimental methods and other results. Supplemental Figure 1: identification of hippocampal neurons and detection of lentivirus transfection efficiency. Supplemental Figure 2: lipopolysaccharide induced mitochondrial α-syn expression in nerve growth factor-differentiated PC12 cells and primary hippocampal neurons. Supplemental Figure 3: activation of the mitochondrial caspase-dependent apoptosis pathway after delayed neurocognitive recovery in vivo and lipopolysaccharide exposure in vitro. [file 5572899.f1.zip › Supplementary Figure 2.pdf]

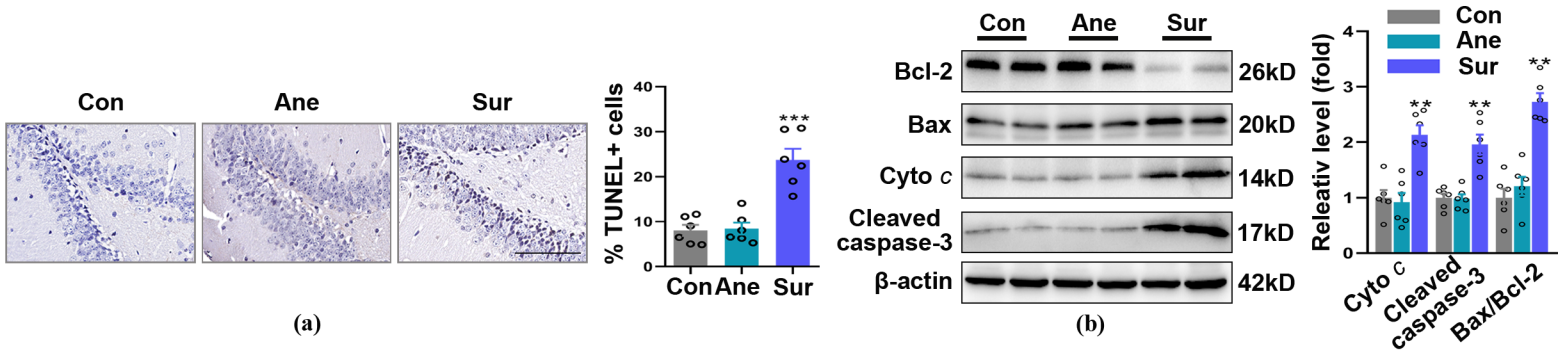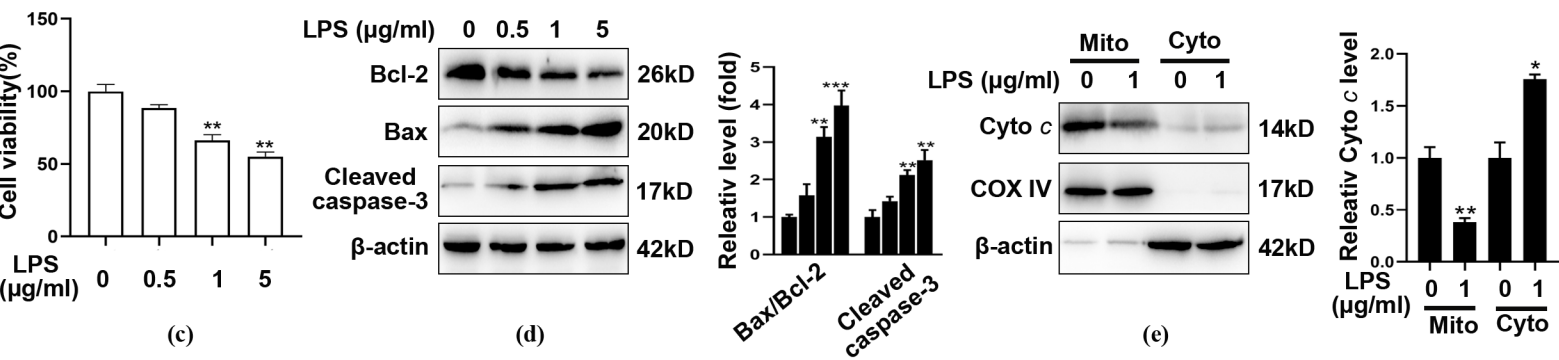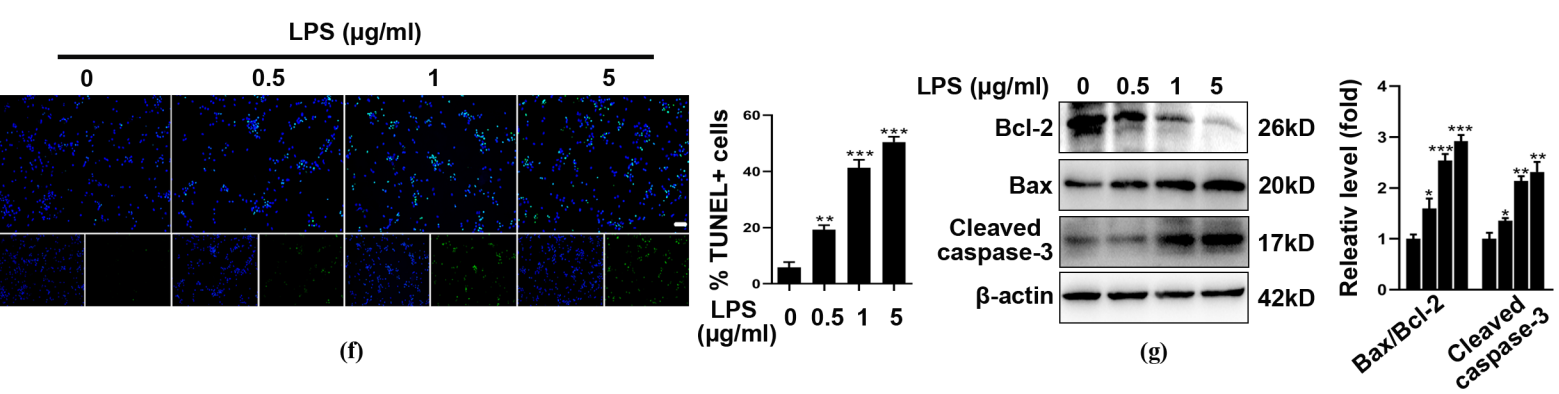

Supplement: Supplementary Materials — include some experimental methods and other results. Supplemental Figure 1: identification of hippocampal neurons and detection of lentivirus transfection efficiency. Supplemental Figure 2: lipopolysaccharide induced mitochondrial α-syn expression in nerve growth factor-differentiated PC12 cells and primary hippocampal neurons. Supplemental Figure 3: activation of the mitochondrial caspase-dependent apoptosis pathway after delayed neurocognitive recovery in vivo and lipopolysaccharide exposure in vitro. [file 5572899.f1.zip › Supplementary Figure 3.pdf]
